# Pre-Osteoblasts Stimulate Migration of Breast Cancer Cells *via* the HGF/MET Pathway

**DOI:** 10.1371/journal.pone.0150507

**Published:** 2016-03-02

**Authors:** Sonia Vallet, Muhammad Hasan Bashari, Feng-Juan Fan, Stefano Malvestiti, Andreas Schneeweiss, Patrick Wuchter, Dirk Jäger, Klaus Podar

**Affiliations:** 1 Department of Medical Oncology, National Center for Tumor Diseases (NCT), University of Heidelberg, Heidelberg, Germany; 2 Department of Pharmacology and Therapy, Faculty of Medicine, Universitas Padjadjaran, Bandung, Indonesia; 3 Department of Obstetrics and Gynecology, University of Heidelberg, Heidelberg, Germany; 4 Department of Medicine V, University of Heidelberg, Heidelberg, Germany; 5 German Cancer Research Center (DKFZ), Applied Tumor Immunity, Heidelberg, Germany; China Medical University, TAIWAN

## Abstract

**Introduction:**

The occurrence of skeletal metastases in cancer, e.g. breast cancer (BC), deteriorates patient life expectancy and quality-of-life. Current treatment options against tumor-associated bone disease are limited to anti-resorptive therapies and aimed towards palliation. There remains a lack of therapeutic approaches, which reverse or even prevent the development of bone metastases. Recent studies demonstrate that not only osteoclasts (OCs), but also osteoblasts (OBs) play a central role in the pathogenesis of skeletal metastases, partly by producing hepatocyte growth factor (HGF), which promotes tumor cell migration and seeding into the bone. OBs consist of a heterogeneous cell pool with respect to their maturation stage and function. Recent studies highlight the critical role of pre-OBs in hematopoiesis. Whether the development of bone metastases can be attributed to a particular OB maturation stage is currently unknown.

**Methods and Results:**

Pre-OBs were generated from healthy donor (HD)-derived bone marrow stromal cells (BMSC) as well as the BMSC line KM105 and defined as ALP^low^ OPN^low^ RUNX2^high^ OSX ^high^ CD166^high^. Conditioned media (CM) of pre-OBs, but not of undifferentiated cells or mature OBs, enhanced migration of metastatic BC cells. Importantly, *HGF* mRNA was significantly up-regulated in pre-OBs *versus* mature OBs, and CM of pre-OBs activated the MET signaling pathway. Highlighting a key role for HGF, CM from HGF-negative pre-OBs derived from the BMSC line HS27A did not support migration of BC cells. Genetically (siMET) or pharmacologically (INCB28060) targeting MET inhibited both HGF- and pre-OB CM- mediated BC cell migration.

**Conclusions:**

Our data demonstrate for the first time a role for pre-OBs in mediating HGF/MET- dependent migration of BC cells and strongly support the clinical evaluation of INCB28060 and other MET inhibitors to limit and/or prevent BC-associated bone metastases.

## Introduction

The metastatic milieu releases specific tissue-homing factors, which determine distinct invasion patterns for regional lymph nodes, lung, liver and bone [1]. In addition, distinct surface receptor profiles support the interaction of tumor cells with the microenvironment at the primary and secondary tumor sites [2, 3]. Mandatory steps in the pathogenesis of skeletal metastases include the intravasation of tumor cells from their primary tumor site into the blood, their extravasation and subsequent invasion of the bone [4, 5]. Despite unprecedented treatment advances in breast cancer (BC), the occurrence of skeletal metastases confers a poor prognosis with 5-year survival rates of less than 10% in patients with bone involvement [6–8]. Therapeutic approaches, which reverse or even prevent the development of bone metastases, are therefore urgently needed. Inhibition of tumor-cell induced signaling sequelae in osteoblasts (OBs) may represent one promising new strategy.

The pathophysiologic role of osteoclasts (OCs) in cancer-associated bone disease is well established. Recent studies also demonstrate a key function of OBs in the development of skeletal metastases. OBs represent a heterogeneous cell pool with respect to their maturation stage, cytokine profile and function. Specifically, OB-lineage cells differ in the spectrum of secreted cytokines, such as CCL2 and RANKL, whose expression levels change during OB maturation [9, 10]. OB progenitor cells, defined by co-expression of RUNX2 and CD166/Activated Leukocyte Cell-Adhesion Molecule (ALCAM), sustain hematopoietic stem cell proliferation and maintenance [11–16].

In the bone, OBs represent the major source of hepatocyte growth factor (HGF), the only known ligand of the receptor tyrosine kinase MET. HGF is a cytokine with pleiotropic functions, including the stimulation of cell proliferation and migration [17–20]. Physiologically, it regulates OC differentiation and supports survival and proliferation of hematopoietic progenitor cells in the bone microenvironment, thereby contributing to bone and hematopoietic homeostasis [18–20]. Moreover, HGF/MET overexpression in solid tumors correlates with disease progression and poor prognosis [21]. Pathophysiologically, HGF is a critical player in the development of skeletal metastases, in BC in particular, by regulating BC cell invasion of the bone [22–25].

The mutual interaction between OBs and tumor cells within the bone milieu has been extensively studied; however, whether a specific subset of osteolineage cells contribute to the pathogenesis of skeletal metastases, *via* the HGF/MET pathway in particular, has not yet been elucidated. In the present study we demonstrate for the first time a key role for ALP^low^ OPN^low^ RUNX2^high^ OSX ^high^ CD166^high^ pre-OBs in HGF/MET-mediated BC cell migration. We thereby highlight the importance of pre-OBs in the pathogenesis of skeletal BC metastases and strongly support a role for targeting MET (e.g. with the specific MET- inhibitor INCB28060) to treat or even prevent BC- associated bone disease.

## Materials and Methods

### Cell lines

All bone marrow samples were acquired from voluntary donors after obtaining written informed consent according to guidelines approved by the Ethics Committee of the Medical Faculty of Heidelberg. This study was approved by the Ethics Committee of the Medical Faculty of Heidelberg (Study No. S-348/2004).

Human mesenchymal stem cells (MSCs) were isolated from human bone marrow aspirates by density-gradient centrifugation, as described previously [26, 27]. Briefly, mononuclear cells were isolated from bone marrow aspirate by density gradient centrifugation using Ficoll-Paque^®^ (GE Healthcare, Munich, Germany) and seeded in plastic culture flasks (Nunc EasYFlasks™ Nunclon™, Thermo Fisher Scientific NUNC A/S, Roskilde, Denmark) at a density of 100,000 mononuclear cells/cm^2^ for 20 days.

The human bone marrow stromal cell line (BMSCs) HS27A was purchased from the American Type Culture Collection (ATCC [28]), KM105 cells were a kind gift of Dr. Kenichi (Chiba University Graduate School of Medicine, Chiba, Japan) [29]. These BMSC lines originate from transfection with the plasmid pSV3gpt and transduction with the human papilloma virus E6/E7, respectively.

The human BC cell line MCF-7 was a kind gift from Dr. P. Beckhove (DKFZ, Heidelberg, Germany) [30], HCC-1954 [31] and MCF-10A cells [32] were from Dr. S. Wiemann (DKFZ, Heidelberg, Germany). MDA-MB231 cells were purchased from the Leibniz Institute/ German Collection of Microorganisms and Cell Cultures, DSMZ (Braunschweig, Germany) [33].

HS27A, KM105, MCF-7, MDA-MB231, HCC-1954 cells were cultured in RPMI 1640 medium supplemented with 10% heat-inactivated fetal bovine serum (FBS) and 1% penicillin/streptomycin. MCF-10A cells were maintained in DMEM/F12 supplemented with 10% heat-inactivated FBS, 1% penicillin/streptomycin, 2,5mg Insulin, 5mg Hydrocortisone, 8μl Cholera toxin, and 10μg hEGF. MSCs were cultivated in commercially available medium (MSCGM; Lonza, Basel, Switzerland).

### Chemicals and reagents

The cell culture media RPMI 1640 and DMEM/F12 were purchased from Gibco, Life Technologies (Grand Island, NY); α-Modified Essential Medium (α-MEM) from Sigma Aldrich (Schnelldorf, Germany). Penicillin/streptomycin was obtained from Gibco, Life Technologies (Grand Island, NY); FBS from PAA Laboratories (Cölbe, Germany). Other media supplements (including insulin, hydrocortisone, cholera toxin, hEGF, β -glycerol phosphate, ascorbic acid, and dexamethasone) were purchased from Sigma Aldrich (Schnelldorf, Germany). Human collagen type I was obtained from BD Biosciences (Heidelberg, Germany).

INCB28060 was purchased from Selleck Chemicals (Munich, Germany) and prepared as a 5 mM stock solution in 100% dimethyl sulfoxide (DMSO) and stored at—80°C [34]. INCB28060 was used at a concentration of 100 nM unless otherwise specified. HGF was purchased from R&D Systems (Minneapolis, MN), diluted in phosphate-buffered saline (PBS) with 1% bovine serum albumin (BSA) and stored at -20°C per manufacturer´s instructions.

Antibodies against human phosphorylated MET and total MET were obtained from Cell Signaling Technology (Boston, MA, USA). Anti-ERK 1/2 antibody was purchased from Santa Cruz Biotechnology (Heidelberg, Germany).

### Osteoblast differentiation of human bone marrow-derived mesenchymal cells and collection of conditioned media

Osteogenic differentiation was performed by plating healthy donor (HD) or immortalized BMSCs to confluence and exposing them to differentiation media (α-MEM with 20% FBS and 1% penicillin/streptomycin supplemented with 2.16 mg/ml β-glycerol phosphate, 0.05 mg/ml ascorbic acid and 10 nM dexamethasone) for up to two weeks. The medium was replaced twice weekly and OBs were analyzed at the specified time-points for cell viability and function, as described previously [35–38]. Briefly, cells were first incubated with AlamarBlue® assay (Invitrogen, Darmstadt, Germany) to assess viability, then fixed and exposed to chromogenic substrate p-nitrophenyl phosphate (Sigma-Aldrich, Schnelldorf, Germany) to quantitate ALP activity. Results are expressed as ALP index (API) by correcting ALP activity for the number of viable cells.

To collect conditioned media (CM), cells at the specified differentiation stage were cultured in α-MEM supplemented with 0,1% FBS and 1% penicillin/streptomycin. After 24 hours the supernatant was collected, centrifuged to remove cell debris and stored at -80°C.

### Cell migration assay

To assess cell migration, BC cells were grown to 70% confluence in 24-well plates [39]. Gaps were introduced by gently scraping the monolayer with a P10 pipette tip and cells were washed three times with PBS to eliminate debris. Cells were then stimulated with CM from differentiating OBs or 50 ng/mL recombinant human HGF in 0,1% α-MEM; 0,1% α-MEM served as negative control. In some experiments cells were simultaneously treated with the MET inhibitor INCB28060. Photographs of the same four fields were taken for each well at the beginning and after 8 hours of incubation. The gap distance was quantified using Image J software[40].

### Cell adhesion assay

Cell adhesion was performed using the Vybrant cell adhesion kit (Molecular Probes, Darmstadt, Germany) according to the manufacturer’s instructions. Briefly, 5x 10^6^ BC cells were labeled with Calcein-AM for 30 min, washed, and resuspended in adhesion media (α-MEM with 0,1% FBS). Following a 3-hour pretreatment with CM or control media, cells were plated in triplicate on type I collagen (2 μg/ml) in the presence of CM; alternatively, cells were directly cultured on monolayers of OB lineage cells. Cells plated in the presence of adhesion media served as negative control. After one hour, unbound cells were removed by gently washing four times with adhesion media. The absorbance of each well was measured with a fluorescence plate reader (Infinite 200 PRO, Tecan, Männedorf, Swiss). In some experiments BC cells were pretreated with INCB28060 for one hour before plating.

### Survival assay

BC cell viability in the presence of OB-derived CM or 0,1% α-MEM was assessed by AlamarBlue® assay according to manufacturer's instructions (Invitrogen, Darmstadt, Germany). Briefly, cells were cultured for three days, then incubated with AlamarBlue for 4 hours and their absorbance measured with a plate reader at 570 nM with wavelength correction at 600 nm (Infinite 200 PRO, Tecan, Männedorf, Swiss).

The cytotoxic effect of INCB28060 on BC cell lines was evaluated by using 3-(4,5-dimethylthiazol-2-yl)-2,5-diphenyl tetrazolium bromide (MTT; Sigma Aldrich, Schnelldorf, Germany). Cells were seeded in 96-well microplates. After 72-hour incubation, 10 μL of MTT solution was added to each well and the plates incubated for 4 hours at 37°C. The optical density was measured in the linear range using a microplate reader at 570 nm with a wavelength correction at 630 nm (Infinite 200 PRO, Tecan, Männedorf, Swiss).

### Quantitative PCR

Gene expression during OB differentiation was analyzed by quantitative real-time PCR. Briefly, RNA was extracted from OBs at the specified differentiation time-points using the RNeasy® Mini Kit (Qiagen, Hilden, Germany), according to the manufacturer's instructions. Oligo-dT primers were used in conjunction with the QuantiTect Reverse Transcription reagents (Qiagen, Hilden, Germany) to synthesize complementary DNA (cDNA), which was processed by real-time quantitative PCR using the QuantiFast SYBR Green (Qiagen, Hilden, Germany) on a LightCycler® 480 detection system (Roche, Mannheim, Germany). Transcript levels were normalized to β-actin and expressed relative to undifferentiated BMSCs. Primers for *RUNX2*, *osterix(OSX)/SP7*, *osteopontin (OPN)* and *HGF* genes were purchased from Qiagen (Hilden, Germany). The primers for human *β-actin* were 5’- CTGGGACGACATGGAGAAAA -3’ (sense) and 5’- AAGGAAGGCTGGAAGAGTGC -3’ (antisense).

### Western blotting

BC cells were cultured to confluence in 6-well plates. After overnight starvation cells were stimulated with HGF 100 ng/ml or CM for one hour, harvested, washed three times with PBS, and lysed in radioimmune precipitation assay (RIPA) lysis buffer (150 mM NaCl, 10 mM Tris pH7.2, 0.1% SDS, 1% Triton X-100, 1% Deoxycholate, 5 mM EDTA) supplemented with Halt Protease and Phosphatase Inhibitor Cocktail (Pierce, Darmstadt, Germany). Samples were then subjected to SDS-PAGE and transferred to nitrocellulose membranes (Amersham, Arlington Heights, IL). After blocking with 5% non-fat dry milk in PBS-Tween®20 buffer, membranes were immunoblotted with the indicated primary antibodies and then with horseradish peroxidase-conjugated secondary antibodies (Santa Cruz Biotechnology, Heidelberg, Germany). Antigen-antibody complexes were detected by enhanced chemiluminescence (Amersham, Arlington Heights, IL). Films were scanned and densitometric analysis performed using the public domain NIH Image J program [40], where indicated.

### Flow cytometry

For determination of the expression of the cell surface marker CD166, cells were harvested with cell dissociation buffer (Invitrogen, Darmstadt, Germany) and suspended in PBS. Following 20 minutes incubation at 4°C with the primary antibody (PE-conjugated mouse CD166 IgG1; eBiosciences, San Diego, CA), cells were washed and analyzed using FACSCANTO II (BD Biosciences, Heidelberg, Germany). Data were analyzed with the FLOWJO program.

### Small interfering RNAs and cell transfection

BC cells were transiently transfected with small interfering RNA (siRNA) siGENOME SMART pools of MET or the non-targeting control (mock) siRNA (Upstate Cell Signaling Solutions/Dharmacon RNA Technologies, Lafayette, CO, USA) using Lipofectamine^®^ 2000 according to the manufacturer's instructions (Invitrogen, Darmstadt, Germany). Nontargeting (mock) siRNA (composed of a pool of four siRNAs, which have been characterized by genome-wide microarray analysis and found to have minimal off-target signatures) served as a control. After 24 hours, cells were used for the migration assays. To ensure gene knockdown, MET expression was verified by western blot assay.

### Statistical analysis

All in-vitro experiments were performed at least in duplicates and repeated three times. All quantitative data were presented as mean ± standard deviation (SD). Statistical comparisons by Student’s two-tailed t test or ANOVA test were considered significant if p < 0.05.

## Results

### The expression pattern ALP^low^ *OSX*^high^ *RUNX2*^high^ CD166^high^ *OPN*^low^ defines the OB subset of pre-OBs

The differentiation of MSCs into OBs proceeds through multiple steps, with stage-defining morphology, surface marker profiles and function. To characterize the function of OB-lineage cells in more detail, we utilized healthy-donor (HD)- derived bone marrow stromal cells (BMSCs) and the immortalized BMSC cell line KM105 [29].

**Fig 1:**
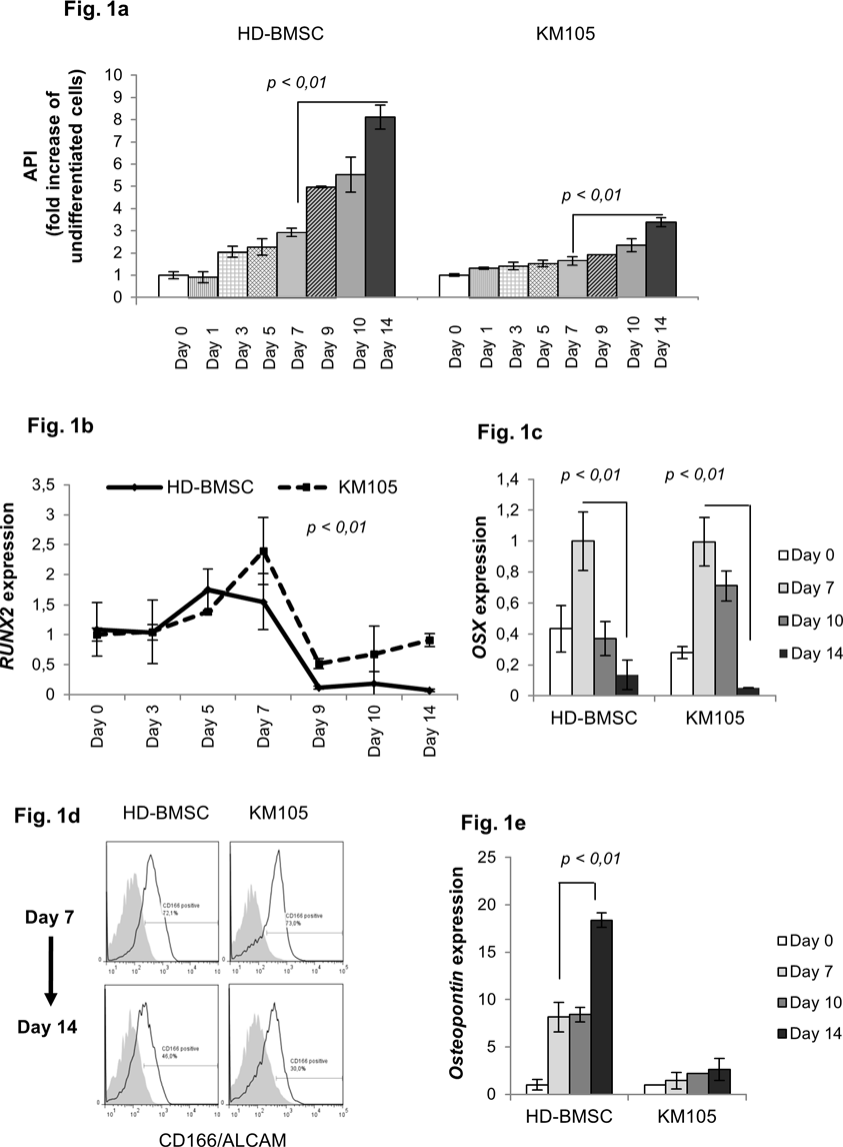
*In-vitro* characterization of early osteoprogenitor cells. (a) *High alkaline phosphatase (ALP) activity characterizes mature osteoblasts (OB)*. ALP activity of healthy donor-derived bone marrow stromal cells (HD-BMSC) and KM105 cells was assessed by ELISA at the specified time-points during osteogenesis and corrected per number of viable cells (ALP index, API). (b) *RUNX2 levels gradually increase during early OB differentiation and decrease with cell maturation*. *RUNX2* was evaluated with quantitative PCR and expressed as fold increase of undifferentiated cells. Statistical analysis was performed with ANOVA test. (c) *Osterix (OSX) levels peak at early steps of differentiation*. *OSX* was evaluated with quantitative PCR and expressed as fold increase of undifferentiated cells. (d) *CD166/ALCAM expression is downregulated by OB maturation in HD-BMSCs and KM105 cells*. Representative results of flow cytometric analysis of the expression of CD166/ALCAM after one (top) and two (bottom) weeks of OB differentiation in HD-BMSC as well as the KM105 cell line are shown. Data represent percentage of positive cells (black line) compared to control (gray curve area). (e) *Osteopontin (OPN) levels increase during OB differentiation*. *OPN* was evaluated with quantitative PCR and expressed as fold increase of undifferentiated cells. Based on the differentiation time-point cells were defined as undifferentiated cells (day 0), pre-OBs (day 7), immature OBs (day 10) and mature OBs (day 14).

HD-BMSCs and KM105 reached OB maturation after 14 days of culture in osteogenic media. Mature OBs were assessed with the API index, which determines the ratio of ALP-expressing cells to viability [35]. An 8-fold increase of ALP activity was observed in HD-BMSC and a 3,3- fold increase in KM105- derived OBs, respectively (Fig 1A) [41]. A subset of osteoprogenitor cells, which we defined as pre-OBs, was generated from HD-BMSCs by exposing them to differentiation media for one week. They were characterized by low ALP activity and high expression of the transcription factor *RUNX2* and *OSX*, both markers of early OB commitment [42, 43]. Specifically, we observed a gradual increase in *RUNX2* RNA levels, which peaked at day 7 and then progressively decreased upon further differentiation (fold- increase of undifferentiated cells of 1,5 in HD-BMSCs, p<0,01). Similarly, *RUNX2* expression increased up to 2,4-fold in KM105 cells after one week of osteogenic differentiation (Fig 1B). As expected, expression of the RUNX2 downstream target *OSX/SP7* peaked at day 7 and then decreased upon further differentiation (Fig 1C). Moreover, we observed an inverse correlation between CD166 expression and cell maturation (72% at day 7 and 46% at day 14, respectively, in HD-BMSCs; and 73% at day 7 and 30% at day 14, respectively, in KM105 cells) (Fig 1D). In contrast, RNA levels of osteopontin (*OPN*), a key modulator of matrix mineralization, significantly increased during osteoblastogenesis [44] (Fig 1E).

Taken together, our *in-vitro* data demonstrate high expression of *RUNX2*, *OSX* and CD166, and low expression of *OPN* and ALP activity in a subset of osteoprogenitor cells derived from HD-BMSCs and KM105 cells after seven days of differentiation, which we defined as pre-OBs. In contrast, mature OBs were defined by low expression of *RUNX2*, *OSX* and CD166, and high *OPN* expression and ALP activity (S1 Fig).

### Pre-osteoblasts stimulate migration and adhesion of metastatic breast cancer cells

**Fig 2:**
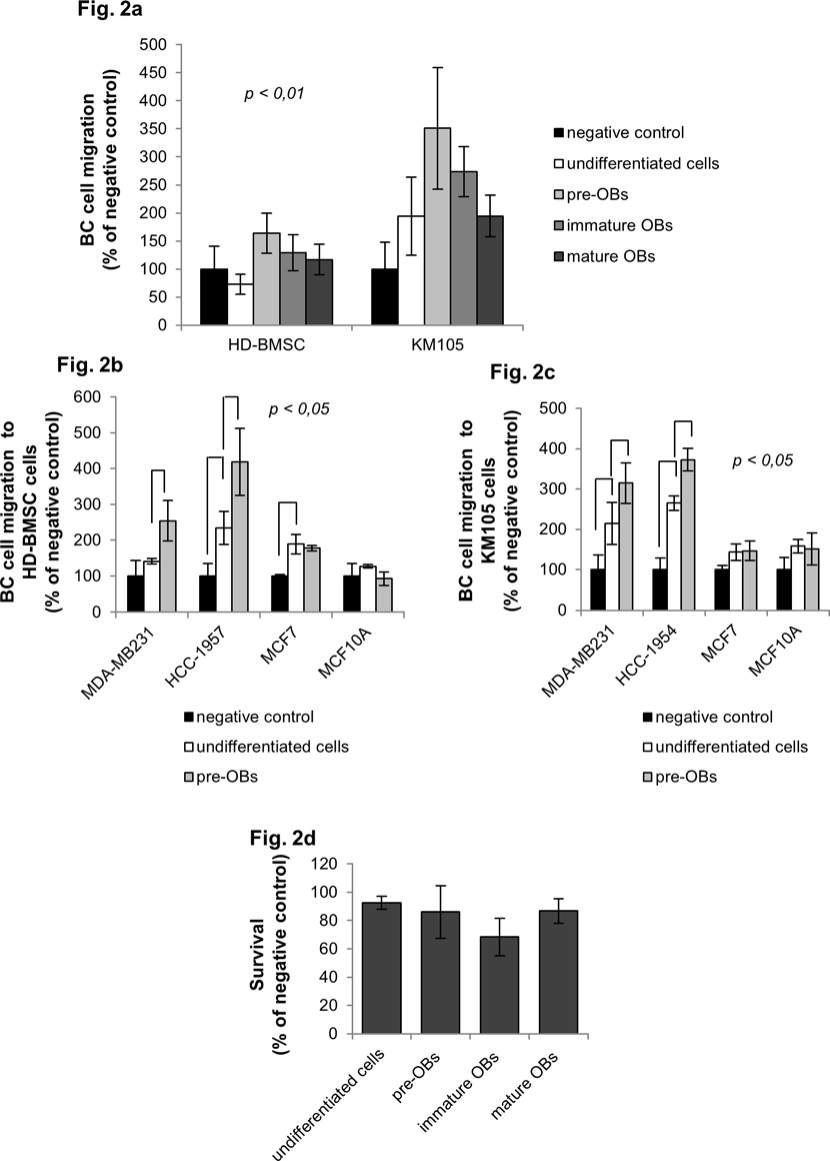
Migration of metastatic BC cell lines to pre-OBs. (a) *Conditioned media (CM) of pre-OBs provide a migratory advantage to MDA-MB231 cells*. The wound healing assay was performed to assess migration of the MDA-MB231 BC cell line in the presence of CM from differentiating OBs derived from HD-BMSCs and KM105 cells. Results are expressed as percentage of negative control. Statistical analysis was performed with the ANOVA test. (b) and (c) *CM of pre-OBs support migration of metastatic BC cells*. Three different BC cell lines, two metastatic (MDA-MB231 and HCC-1954), and one invasive, but non-metastatic (MCF7); and one benign breast cell line (MCF10A), were subjected to the wound healing assay in the presence of CM from HD-BMSC- (b) or KM105-derived (c) pre-OBs or undifferentiated cells. Results are expressed as percentage of negative control. (d) *CM of pre-OBs do not affect BC cell survival*. MDA-MB231 cells were cultured in the presence of CM from KM105-derived osteolineage cells for three days. Cell survival was assessed with AlamarBlue assay.

We next evaluated the ability of pre-OBs to support migration and adhesion of a panel of BC cell lines with different metastatic and invasive properties. As shown in Fig 2A, CM of pre-OBs but not CM from undifferentiated cells or mature OBs increased migration of the metastatic BC cell line MDA-MB231 (1,6-fold increase in HD-BMSC-derived pre-OBs *versus* 0,7- and 1,1-fold increase in undifferentiated HD-BMSC and HD-BMSC-derived mature OBs, respectively; and 3,5- fold increase in KM105-derived pre-OBs *versus* 1,93- and 1,94-fold increase in undifferentiated KM105 cells and KM105-derived mature OBs, respectively; p<0,01). Similar results were obtained in the metastatic BC cell line HCC-1954 (1,8- and 1,4-fold increase of migration in the presence of CM from HD-BMSC-derived and KM105-derived pre-OBs *versus* undifferentiated cells, Fig 2C and 2C). In contrast, CM from HD-BMSC- and KM105-derived pre-OBs did not modulate migration of the non-metastatic BC cell line MCF7 and the benign breast cell line MCF10A (Fig 2B and 2C). Importantly, no effect on cell survival was observed when BC cells were cultured in the presence of CM from KM105-derived OB-lineage cells for up to three days (Fig 2D).

Seeding of tumor cells into the metastatic bone niche is associated with the interaction of tumor cells and OBs. Whether OB lineage cells differ in their adhesion strength to BC cells is currently unknown. We therefore next investigated BC cell adhesion to OB-lineage cells. Cell adhesion of MDA-MB231 and HCC-1954 to KM105-derived pre-OBs was strongly increased when compared to undifferentiated cells or mature OBs (59% of MDA-MB231 cells adhered to KM105-derived pre-OBs *versus* 34% to undifferentiated cells *versus* 37% to mature OBs, p<0,05, S2A Fig; and 44% of HCC-1954 cells adhered to KM105-derived pre-OBS *versus* 24% to undifferentiated cells; S2B Fig). In contrast, we did not observe changes in adhesion of the non-metastatic BC cell line MCF7 and the benign breast cell line MCF10A to pre-OBs *versus* undifferentiated cells (S2B Fig). To evaluate the role of soluble factors in mediating cell adhesion, we next plated MDA-MB231 cells on type I collagen in the presence of CM from KM105-derived OB-lineage cells. As shown in S2C Fig, CM of pre-OBs did not provide any advantage in terms of BC cell adhesion to collagen. These data suggest that different mechanisms regulate BC cell migration into and BC cell adhesion to pre-OBs in the bone niche. Ongoing studies aim to unravel the surface molecules responsible for BC cell adhesion to pre-OBs.

Taken together, our data show for the first time that CM from pre-OBs enhance migration of metastatic BC cells and that metastatic BC cells preferentially adhere to pre-OBs.

### *HGF* is expressed by pre-OBs and mediates tumor cell migration

**Fig 3:**
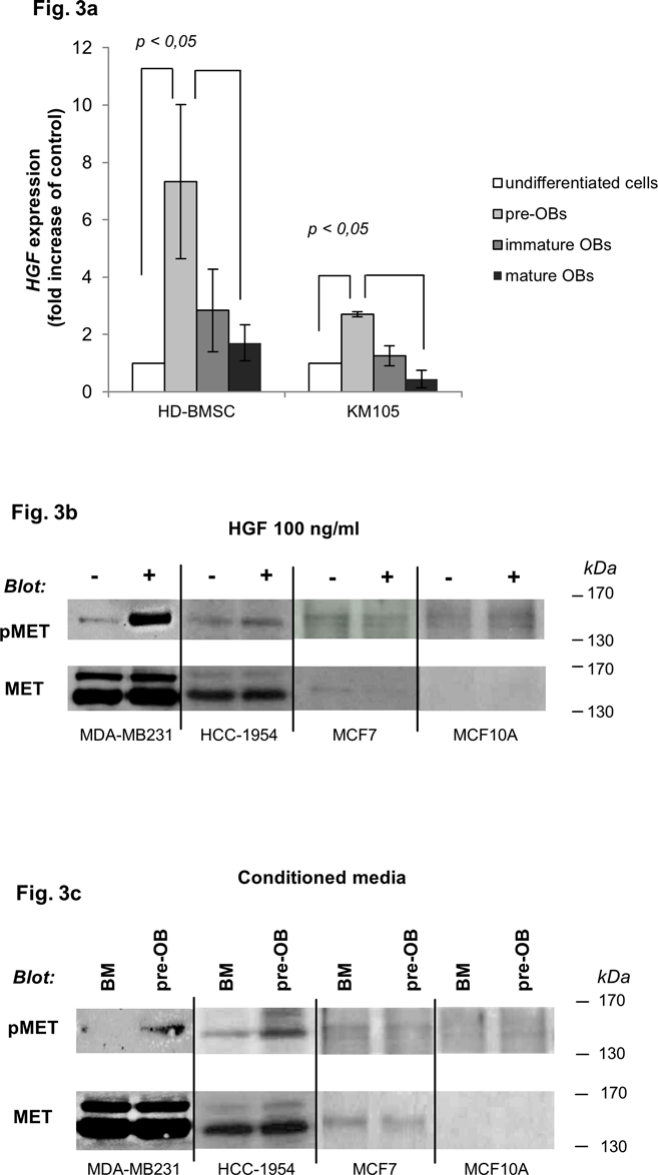
HGF is upregulated in pre-OBs and activates MET signaling in BC cells. (a) *HGF gene levels are upregulated in healthy-donor derived bone marrow stromal cells (HD-BMSCs) and KM105-derived pre-OBs*. mRNA expression of *HGF* was analyzed by quantitative PCR and expressed as fold increase of undifferentiated cells. (b) *Exogenous HGF triggers MET phosphorylation in MDA-MB231 and HCC-1954 cells*. MDA-MB231, HCC-1954 and MCF7 BC cells as well as the benign breast cell line MCF10A were challenged with HGF for one hour. Phosphorylation of MET and total MET were determined by western blot analysis. (c) *Conditioned media (CM) of pre-OBs activate MET signaling in MDA-MB231 and HCC-1954 cells*. MDA-MB231, HCC-1954 and MCF7 BC cells as well as the benign breast cell line MCF10A were stimulated with CM of KM105-derived pre-OBs or undifferentiated cells for one hour. Phosphorylation of MET and total MET were determined by western blot analysis.

Migration of tumor cells to target organs is driven by cytokines, including HGF [23]. Within the bone microenvironment OBs represent the main source of HGF [18, 19]. Whether HGF production differs among OB-lineage cells is unknown. Using gene expression analysis our data show that *HGF* mRNA is up-regulated in pre-OBs derived from HD-BMSCs and the KM105 cell line (7,3- and 2,7- fold increase, respectively, p< 0,05, Fig 3A), when compared to undifferentiated cells and mature OBs. Importantly, exposure to HGF and CM of KM105-derived pre-OBs, but not CM of undifferentiated cells upregulated MET phosphorylation in MDA-MB-231 cells and HCC-1954 cells, respectively. In contrast, stimulation with HGF or CM of KM105-derived pre-OBs in MCF7 and MCF10A cells with low/no MET expression did not increase MET phosphorylation (Fig 3B and 3C).

**Fig 4:**
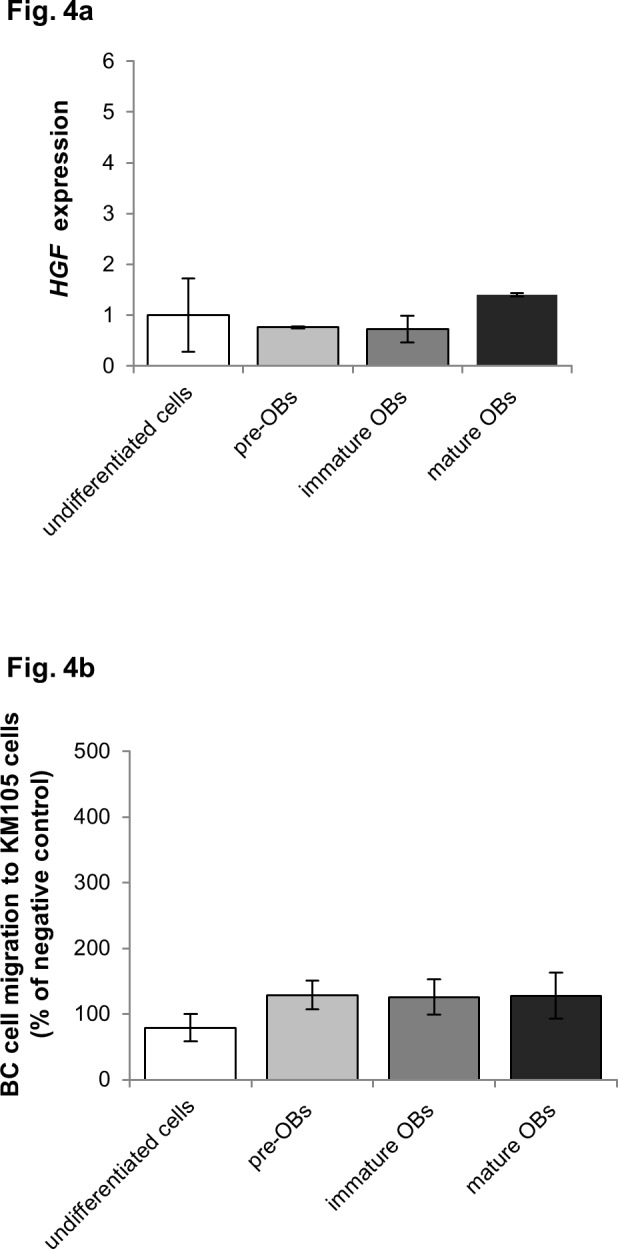
CM of pre-OBs derived from HGF-negative HS27 cells do not induce BC cell migration. (a) *Low HGF gene levels in HS27A-derived pre-OBs*. mRNA expression of *HGF* was analyzed by quantitative PCR and expressed as fold increase of undifferentiated cells. (b) *CM of HS27A-derived pre-OBs do not induce BC cell migration*. Wound healing assay was performed to assess migration of the MDA-MB231 BC cell line in the presence of CM from HS27A-derived osteolineage cells. Results are expressed as percentage of negative control.

Previous studies reported low expression levels of HGF in the human BMSC line HS27A [45]. We next evaluated BC cell migration triggered by CM of HS27A-derived pre-OBs. Concordantly to the low RNA expression levels of *HGF* in HS27A-derived pre-OBs (Fig 4A), CM from HS27A-derived pre-OBs did not modulate migration of the metastatic BC cell line MDA-MB231 (Fig 4B). Taken together, our data strongly support a key role of HGF in pre-OB- induced migration of metastatic BC cells.

### Inhibition of MET signaling overcomes the migratory advantage of BC cells stimulated with CM of pre-OBs

Based on these data we hypothesized that HGF/MET may represent a potential therapeutic target to overcome skeletal metastases. Several inhibitors of the HGF/MET signaling are currently undergoing clinical evaluation in a variety of solid cancers [46]. INCB28060, in particular, is an orally available specific small molecule inhibitor of MET with a favorable safety profile and promising preliminary clinical data [34].

**Fig 5:**
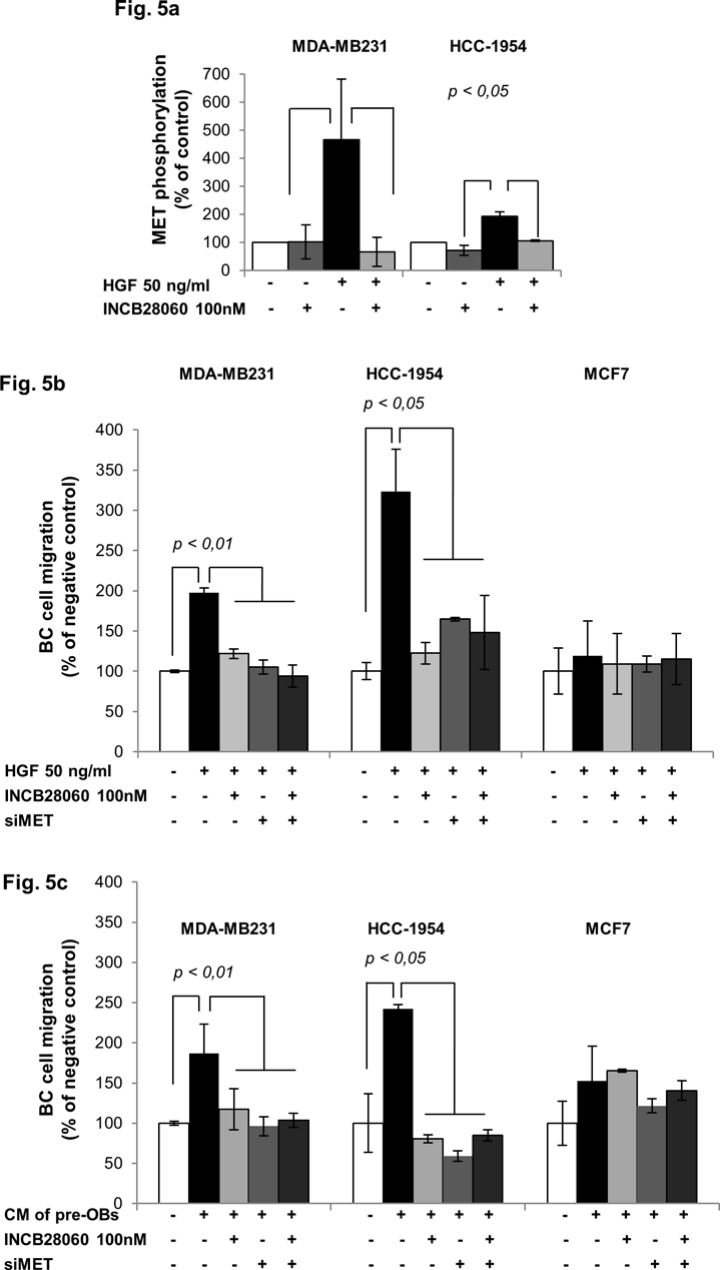
MET inhibition overcomes HGF and pre-OB-induced migration of BC cells. (a) *MET inhibition by INCB28060 prevents HGF-induced MET phosphorylation in MDA-MB231 and HCC-1954 cells*. After one hour preincubation with INC2B8060, BC cells were stimulated with HGF for one hour. Phosphorylation of MET and total MET were determined by western blot and quantified by densitometric analysis. Results are expressed as percentage of unstimulated and untreated cells (negative control). *(d) MET inhibition by INC2B8060 or siMET prevent HGF-induced migration of BC cells*. 8-hour migration of MDA-MB231, HCC-1954 and MCF7 cells transfected with mock or siMET in the presence of HGF with or without INCB28060 was evaluated by a wound healing assay. (e) *MET inhibition impairs the migration advantage of MDA-MB231 BC cells provided by pre-OBs*, *but not undifferentiated cells*. After one hour preincubation with INCB28060, MDA-MB231 BC cells transfected with mock or siMET were exposed to CM of KM105-derived pre-OBs or undifferentiated cells and migration was assessed by wound healing assay. Percentage of negative control is shown. (f) *MET inhibition impairs the migration advantage of HCC-1954 BC cells provided by pre-OBs*. After one hour preincubation with INCB28060, BC cells transfected with mock or siMET were exposed to CM of KM105-derived pre-OBs in the presence of INCB28060 to assess migration by a wound healing assay. Percentage of negative control is shown.

In order to evaluate the role of HGF/MET- signaling in BC cell migration we next utilized INCB28060 as well as siRNA-mediated knockdown of MET gene (siMET). Treatment with 100 nM INCB28060 significantly inhibited HGF-triggered MET phosphorylation in MDA-MB231 and HCC-1954 cells (Fig 5A). INCB28060 or siMET suppressed HGF-induced migration of both MDA-MB231 and HCC-1954 cells (Fig 5B, S3 Fig). Moreover, MET inhibition by INCB28060 or siMET overcame the migratory advantage triggered by CM of KM105-derived pre-OBs in MDA-MB231 and HCC-1954 BC cells (Fig 5C), but had no effect on migration induced by the CM of undifferentiated cells (data not shown). As expected, treatment with INCB28060 or siMET had no effect on MET-negative MCF7 cell migration (Fig 5C). Importantly, co-treatment with INCB28060 and siMET did not further increase the inhibitory effect of single INCB28060 or siMET treatment, confirming that MET is their common target. Finally, BC cell adhesion to pre-OBs was not inhibited by INCB28060 (S4 Fig), further confirming the independence of BC cell adhesion from soluble factors, e.g. HGF. Importantly, a cytotoxic effect of INCB28060 on the BC cell lines MDA-MB231, HCC-1954, MCF7 and on the benign breast cell line MCF10A was observed only after 72 hours of treatment with concentrations above 20 μM (S5 Fig).

Taken together, inhibition of the HGF/MET signaling pathway impairs the migration advantage provided by pre-OBs to metastatic BC cells, thus supporting the use of MET inhibitors such as INCB28060 for the treatment and potential prevention of skeletal metastases in BC.

## Discussion

**Fig 6:**
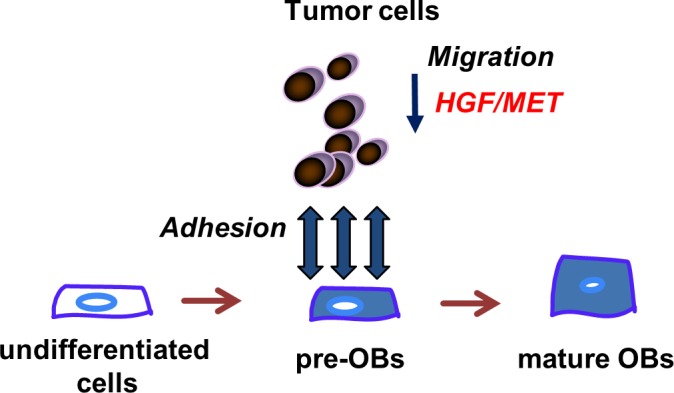
Schematic representation of the interactions between pre-OBs and tumor cells. Pre-OBs enhance migration of breast cancer (BC) cells via activation of the HGF/MET pathway. In addition, metastatic BC cells preferentially adhere to pre-OBs.

Intravasation, extravasation, and subsequent invasion of tumor cells from the primary tumor site to distant organs are mandatory steps in the onset of metastases [4]. In 3D-models tumor cell colonization of the bone is regulated by OBs at different maturation stages. Specifically, tumor cell proliferation is predominantly supported by osteoprogenitor cells and invasion by mature OBs [47]. Here, we demonstrate for the first time that a subset of osteolineage cells, pre-OBs, modulates migration and adhesion of BC cells (Fig 6).

Mature OBs are well characterized cells. In contrast, the identification of their precursors remains a challenge due to the absence of reliable surface markers. CD166 is an adhesion molecule involved in a wide range of physiologic and pathologic events [11–14]. In the bone microenvironment the co-expression of CD166 and *RUNX2* defines a subset of osteoprogenitor cells involved in the maintenance of hematopoietic stem cells [15, 16]. Here, we identified HD-BMSC and BMSC line KM105-derived pre-OBs by the simultaneous expression of *RUNX2*, *OSX* and CD166 and the absence of ALP activity. In contrast, undifferentiated cells expressed only CD166; and mature OBs expressed high *OPN* levels and were characterized by high ALP activity.

Recent studies suggest that tissue-specific signaling drives cancer cell clones with a permissive receptor profile to the target organ [1–3]. HGF is a major component of the OB-derived hematopoietic activity within the endosteal niche [19, 48], and tumor cells typically localize close to the endosteum [49, 50]. Indeed, the HGF/MET pathway plays a critical role in the development of skeletal metastases, in particular in BC [23, 46, 51]. Here, we show for the first time that pre-OBs are the main source of HGF. Moreover, our data demonstrate that HGF mediates MET-dependent migration of metastatic BC cells (Fig 6).

Human BMSC lines KM105 and HS27A promote hematopoiesis *via* surface interactions; but differ in their cytokine profile (e.g. HGF) [45], since only KM105-derived supernatant maintains hematopoietic stem cells [28, 29]. Differences in HGF levels between KM105 and HS27A explain, at least in part, the discrepant effect on BC cell migration. Indeed, consistent with a key role of HGF in BC cell migration, our results show that CM of HGF-producing KM105-derived pre-OBs but not HS27A-derived pre-OBs, which lack HGF production, trigger BC cell migration. Moreover, CM of KM105-derived pre-OBs and HD-BMSC-derived pre-OBs or exogenous HGF triggered cell migration of MET expressing MDA-MB231 and HCC-1954 cells, but not MCF7 and MCF10A cells, which lack MET expression.

Based on these data we hypothesized that HGF/MET may represent a potential therapeutic target to overcome bone metastases. Indeed, our results show that INCB28060 as well as siRNA-mediated knockdown of MET block pre-OB-induced migration of BC cells without compromising their survival. Moreover, our data demonstrate an increase of BC cell adhesion to pre-OBs. However, in contrast to migration, soluble factors derived from osteolineage cells had no effect on the adhesion of BC cells. Consequently, INCB28060 lacked to inhibit BC cell adhesion to pre-OBs (S4 Fig), while both fixed pre-OBs as well as non-fixed pre-OBs induced BC cell adhesion (data not shown). Ongoing studies aim to identify surface molecules responsible for BC cell adhesion to pre-OBs.

Of interest, in agreement with previous findings from Mercer et al and Mendoza-Villanueva et al., our results additionally indicate that BC cells impair OB maturation. [52, 53] Specifically, CM from MDA-MB231 BC cells upregulated early markers of osteogenesis, such as OSX, and inhibited ALP activity in late stage OBs (data not shown and S5 Fig). Similarly, Kassen et al. report on the expansion of osteoprogenitor cells in a murine model of myeloma-induced bone disease [54]. We currently seek to delineate molecular mechanisms by which BC cells impair OB maturation in more detail.

Taken together, this study shows for the first-time that pre-OBs mediate migration of BC cells by activating the HGF/MET pathway. Indeed, our in vitro data strongly support the clinical evaluation of INCB28060 and other MET inhibitors to limit and/or prevent BC-associated bone metastases.

## Supporting Information

S1 Fig Schematic representation of *in-vitro* markers for osteogenic differentiation. Pre-osteoblasts (pre-OBs) (after one week of *in-vitro* differentiation) express high *RUNX2*, *OSX* and CD166 levels and show low expression of *OPN* and alkaline phosphatase (ALP) activity. In contrast, low *RUNX2*, *OSX*, *OPN* expression and low ALP activity are observed in undifferentiated cells (at day 0), and low *RUNX2*, *OSX* and CD166 levels in mature OBs (two weeks of differentiation).
(PDF)

S2 Fig Adhesion of metastatic breast cancer (BC) cell lines to pre-osteoblasts (pre-OB). (a) *Adhesion of MDA-MB231 cells is enhanced by KM105-derived pre-OBs*. Percentage of adherent MDA-MB231 cells to KM105-derived osteolineage cells is shown. (b) *Adhesion of metastatic BC cell lines is stimulated by KM105-derived pre-OBs*. Adhesion of four different BC cell lines to undifferentiated cells and pre-OBs derived from KM105 is shown. (c) *Conditioned media (CM) of pre-OBs do not influence adhesion of MDA-MB231 cells to collagen*. MDA-MB231 cells were pre-incubated with CM of KM105-derived osteolineage cells for three hours and then plated on type I collagen. Percentage of adherent cells is shown.
(PDF)

S3 Fig MET expression is downregulated by siRNA treatment. BC cells were transfected with mock or siMET and MET was determined by western blot. ERK1/2 served as loading control.
(PDF)

S4 Fig MET inhibition does not influence adhesion of BC cells to KM105-derived pre-OBs. After one hour preincubation with INCB28060, BC cells were plated on KM105-derived pre-OBs or undifferentiated cells for one hour.
(PDF)

S5 Fig Cytotoxic effects of INCB28060 are observed at high concentrations. BC cell lines (MDA-MB231, HCC-1954 and MCF7) as well as benign breast cell line MCF10A were treated with the MET inhibitor INCB28060 for 72 hours. Cytotoxicity was assessed with MTT assay.
(PDF)

S6 Fig Conditioned media (CM) of MDA-MB-231 cells inhibit alkaline phosphatase (ALP) activity during osteogenesis. KM105 cells were exposed to OB differentiation media for 7 or 10 days in the presence of CM derived from MDA-MB231 cells. ALP activity was assessed by ELISA at the specified time-points and corrected per number of viable cells (ALP index, API).
(PDF)
