# Supplementary figures and images for: Pre-Osteoblasts Stimulate Migration of Breast Cancer Cells via the HGF/MET Pathway
Source: PLoS One. 2016 Mar 2;11(3):e0150507. doi: 10.1371/journal.pone.0150507 (PMC4774929; doi:10.1371/journal.pone.0150507)

S1 Fig

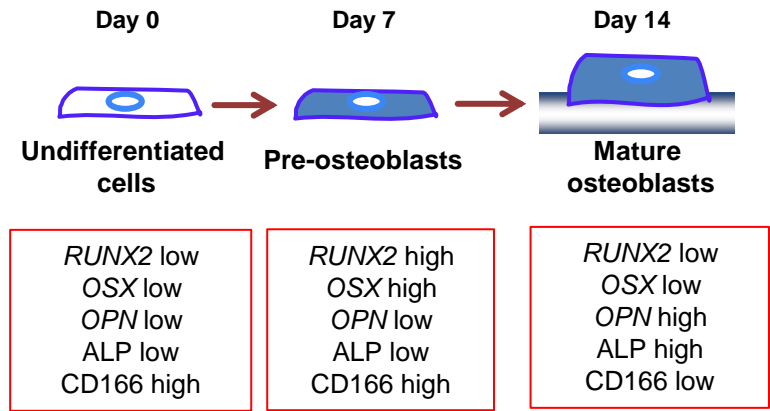

Supplement: S1 Fig — Pre-osteoblasts (pre-OBs) (after one week of in-vitro differentiation) express high RUNX2, OSX and CD166 levels and show low expression of OPN and alkaline phosphatase (ALP) activity. In contrast, low RUNX2, OSX, OPN expression and low ALP activity are observed in undifferentiated cells (at day 0), and low RUNX2, OSX and CD166 levels in mature OBs (two weeks of differentiation). (PDF) [file pone.0150507.s001.pdf]

**S2a Fig**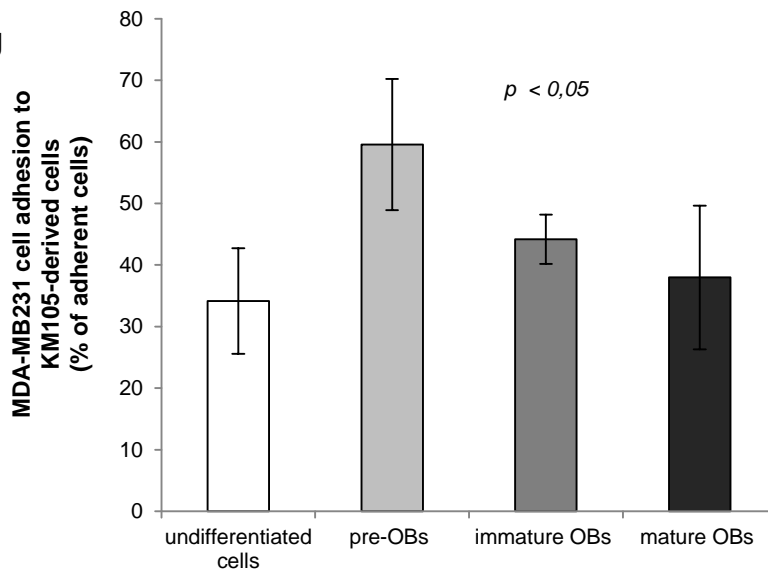**S2b Fig**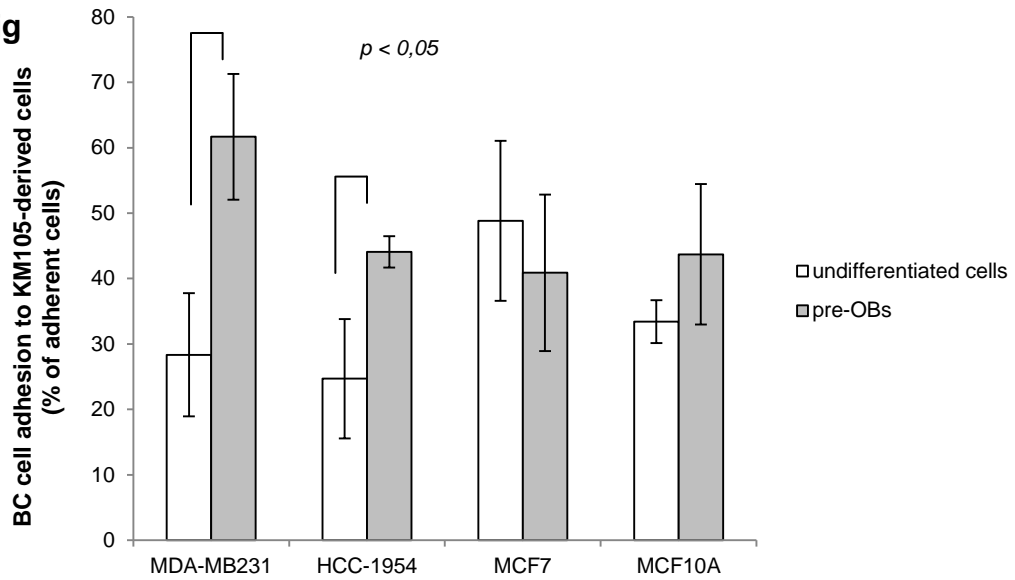**S2c Fig**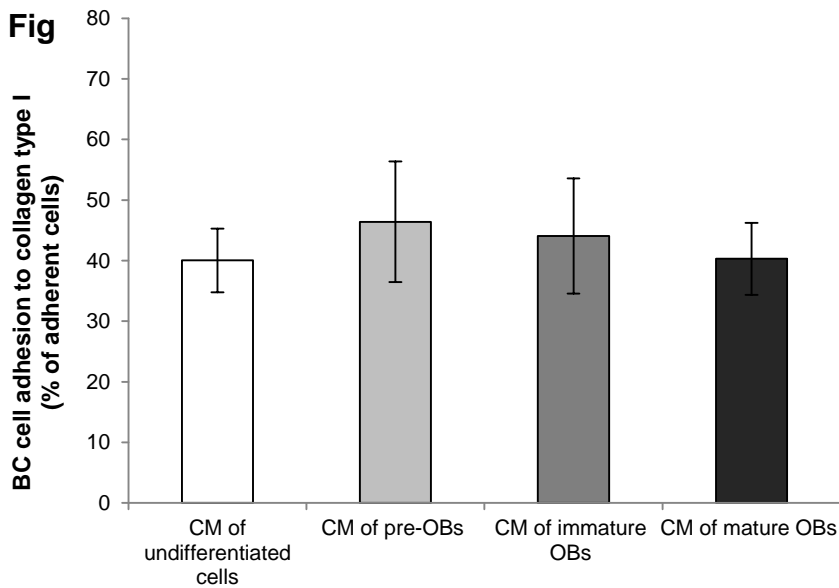

Supplement: S2 Fig — (a) Adhesion of MDA-MB231 cells is enhanced by KM105-derived pre-OBs. Percentage of adherent MDA-MB231 cells to KM105-derived osteolineage cells is shown. (b) Adhesion of metastatic BC cell lines is stimulated by KM105-derived pre-OBs. Adhesion of four different BC cell lines to undifferentiated cells and pre-OBs derived from KM105 is shown. (c) Conditioned media (CM) of pre-OBs do not influence adhesion of MDA-MB231 cells to collagen. MDA-MB231 cells were pre-incubated with CM of KM105-derived osteolineage cells for three hours and then plated on type I collagen. Percentage of adherent cells is shown. (PDF) [file pone.0150507.s002.pdf]

**S3 Fig**

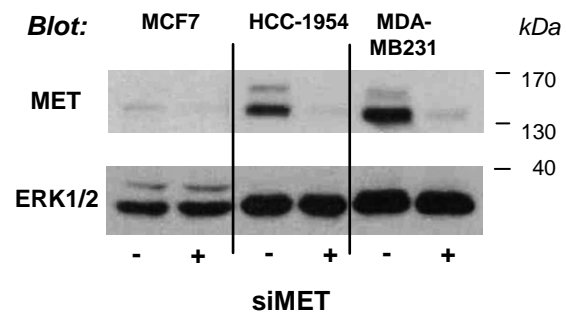

Supplement: S3 Fig — BC cells were transfected with mock or siMET and MET was determined by western blot. ERK1/2 served as loading control. (PDF) [file pone.0150507.s003.pdf]

**S4 Fig**

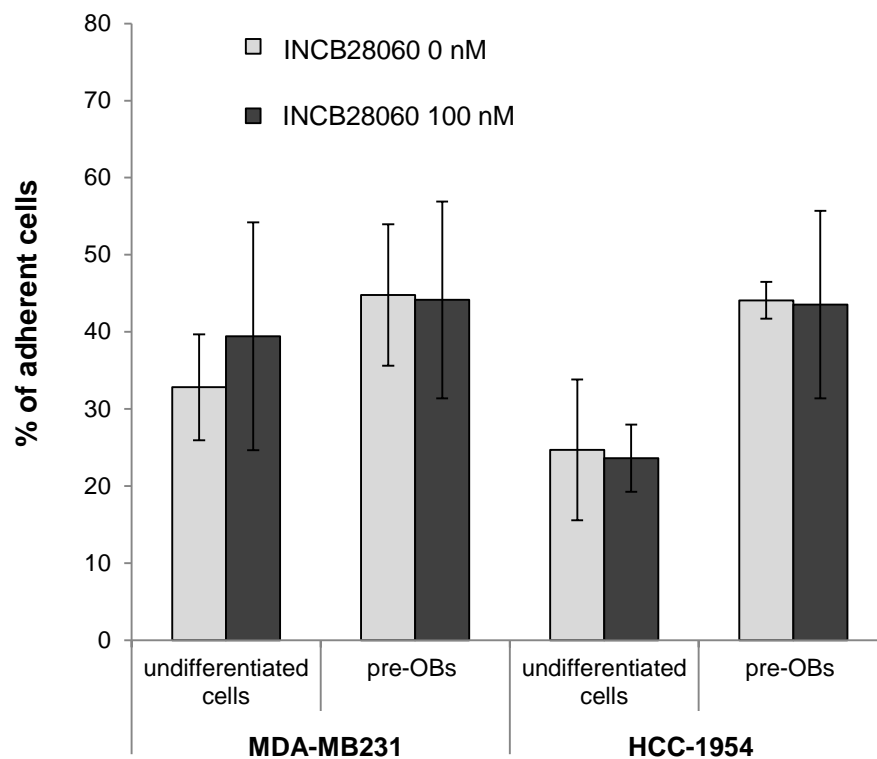

Supplement: S4 Fig — After one hour preincubation with INCB28060, BC cells were plated on KM105-derived pre-OBs or undifferentiated cells for one hour. (PDF) [file pone.0150507.s004.pdf]

**S5 Fig**

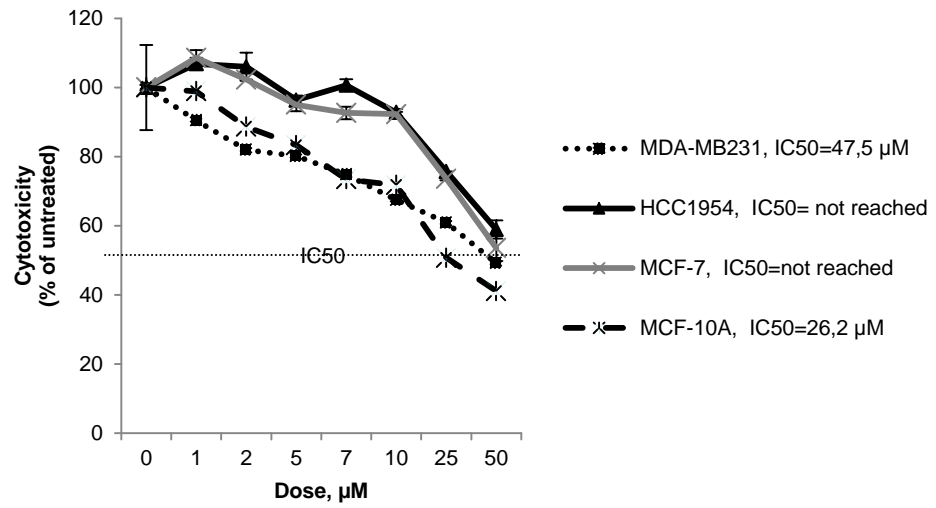

Supplement: S5 Fig — BC cell lines (MDA-MB231, HCC-1954 and MCF7) as well as benign breast cell line MCF10A were treated with the MET inhibitor INCB28060 for 72 hours. Cytotoxicity was assessed with MTT assay. (PDF) [file pone.0150507.s005.pdf]

**S6 Fig**

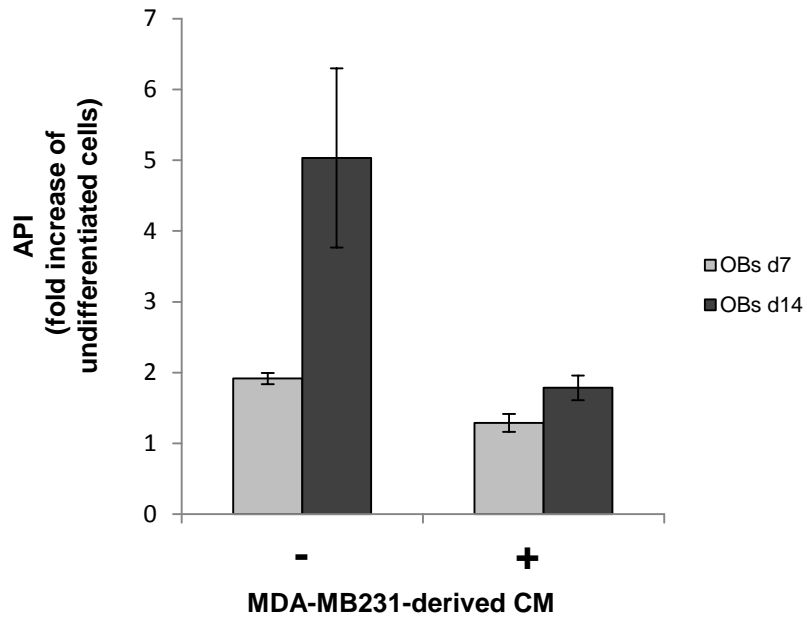

Supplement: S6 Fig — KM105 cells were exposed to OB differentiation media for 7 or 10 days in the presence of CM derived from MDA-MB231 cells. ALP activity was assessed by ELISA at the specified time-points and corrected per number of viable cells (ALP index, API). (PDF) [file pone.0150507.s006.pdf]
